# Correction potential and outcome of various surgical procedures for hallux valgus surgery: a living systematic review and meta-analysis

**DOI:** 10.1007/s00402-024-05521-0

**Published:** 2024-09-09

**Authors:** S. Ettinger, F. T. Spindler, M. Savli, D.A.F. Scientific Committee, Christina Stukenborg-Colsman, Sabine Ochman, Stefan Rammelt, Hans Polzer, Natalia Gutteck, Norbert Harrasser, Christian Plaaß, Sebastian F. Baumbach

**Affiliations:** 1 University Hospital for Orthopaedics and Trauma Surgery, Pius-Hospital Oldenburg, Georgstrasse 12, 26121 Oldenburg, Germany; 2 Department of Orthopaedics and Trauma Surgery, Musculoskeletal University Center Munich (MUM), University Hospital, LMU Munich, Ziemssenstraße 5, 80336 Munich, Germany; 3 Biostatistik and Consulting Savli, Leutschenbachstrasse 95, 8050 Zurich, Switzerland; 4 Deutsche Assoziation für Fuß und Sprunggelenk e.V., Strasse des 17. Juni 106-108, 10623 Berlin, Germany

**Keywords:** Meta-analysis, Living systematic review, Hallux valgus, Forefoot deformity

## Abstract

**Introduction:**

More than 100 surgical techniques are described for hallux valgus (HV) correction, but the most appropriate technique remains debatable. The aim of this study was to develop and conduct a “living systematic review” for the outcome of surgically treated HV.

**Materials and methods:**

The “living systematic review” was conducted per the PRISMA-P and PICOS guidelines and is the basis for the German AWMF S2e guideline “Hallux valgus” (033-018). Four common databases and the grey-literature were searched. Eligible were studies on adult patients comparing either two different primary surgical interventions or the same primary surgical intervention for different hallux valgus severities. The main outcome parameters were the osseous correction potential and the patient rated outcome.

**Results:**

Out of 3022 studies, 46 studies (100 arms) were included. The meta-analysis included 31 studies (53 arms). The IMA (1933 procedures) improved on average by 7.3°, without significant group differences. The HVA (1883 procedures) improved on average by 18.9°, with significantly better results for third generation MIS (21.2°). The AOFAS (1338 procedures) improved on average by 33.8 points without significant group differences. The meta-regression revealed constant AOFAS scores over time. 69%/39% of the correction potential for the IMA/HVA could be explained by the preoperative values and 82% of the AOFAS improvement by the preoperative AOFAS scores.

**Conclusion:**

Open and minimally invasive techniques are powerful tools to correct hallux valgus deformity. Third generation MIS procedures revealed a possible superiority for the correction of the HVA. The AOFAS improvement appeared to be constant over time.

**Level of evidence:**

Level I; living systematic review and meta-analysis of prospective comparative studies (level II) and randomized controlled trials (level I).

**Supplementary Information:**

The online version contains supplementary material available at 10.1007/s00402-024-05521-0.

## Introduction

Hallux valgus (HV) is one of the most common forefoot deformities, characterized by a lateral deviation of the great toe and a medial deviation of the first metatarsal bone [1]. After failed conservative treatment, surgery can realign the first ray [2]. According to Mann and Coughlin, HV can be divided into three grades based on the intermetatarsal angle (IMA) and hallux valgus angle (HVA) measured on dorso-plantar weightbearing radiographs: mild (IMA < 11°, HVA < 20°), moderate (IMA 11–16°, HVA 20–40°), and severe (IMA > 16°, HVA > 40°) [1]. Overall, more than one hundred surgical techniques are described for HV correction. These can be grouped into distal-, diaphyseal-, or proximal osteotomies, and arthrodesis.

More recently, percutaneous and minimally invasive surgical techniques have gained increasing recognition, because of the potential advantages of better range of motion (ROM) and less soft tissue trauma [3, 4]. Until now, three generations of minimally invasive HV surgery have been developed. The first generation, reported in 1991, was the Reverdin Isham technique, which was performed without internal fixation [5]. The second generation is the Bösch osteotomy [6], using Kirschner wires for fixation [7]. The minimally invasive chevron and akin (MICA) described by Vernois and Redfern represents the third generation, using screws for fixation [8–10]. Recently, an adaptation variant of this technique was described, using a metaphyseal extra-articular transverse and akin osteotomy (META) [11].

Although the type of surgical procedure is traditionally chosen according to the severity of the HV deformity [10, 12], the decision remains up to the surgeon’s preference [13]. Up to date, a consensus on the most appropriate treatment approach is still missing. Traditionally, mild deformities are addressed by distal osteotomies, moderate deformities by shaft osteotomies, and severe deformities by proximal osteotomies or arthrodesis [14].

The aim of this study was to (1) develop a “living systematic review” for the outcome of surgically treated hallux valgus and to (2) apply and analyze this search strategy for the timeframe from Jan 1st 2012 to Jan 31st 2023. The “living systematic review” is the basis for regular (5-year interval) updates of the German AWMF S2e guideline “Hallux valgus” (033-018). The two primary outcome variables are the osseous correction and the patient rated outcome.

## Materials and methods

The living systematic review is the basis for regular (5-year interval) updates of the German AWMF S2e guideline “Hallux valgus” (033-018). The herein developed and applied systematic review was conducted per the Preferred Reporting Items for Systematic Reviews and Meta-Analyses (PRISMA-P) guidelines [15]. The study was a-priori registered at Prospero (CRD42021261490). The PICOS criteria were changed throughout the review process. Due to the large number of studies available, only original comparative studies, prospective or randomized controlled trials, were included. This amendment was reported to Prospero.

### Search strategy

MEDLINE (PubMed), Scopus, Central, and EMBASE were searched from inception to Jan 31st 2023. For the current analysis, studies prior to Jan 1st 2012 were excluded. A grey literature search was performed in both Scopus and EMBASE including conference proceedings. Additionally, all references of the studies included were hand-searched. The search strategy comprised of the following principal strategies: Hallux valgus AND Surgery. The detailed search strategy is outlined in Supplement 1.

The in- and exclusion criteria were designed according to the Population, Intervention, Comparison, Outcomes and Study (PICOS) criteria and are summarized in Table 1 [16].

**Table 1:** PICOS criteria

Population	Adult patients treated for a primary hallux valgus deformity. Adult was defined as ≥ 18 years
Intervention	Any primary surgical procedure for a hallux valgus deformity
Comparison	Another primary surgical procedure for a hallux valgus deformity OR the same surgical procedure comparing different stages of deformity
Outcomes	Any objective outcome, including functional outcome, patient reported outcome, return to work / sports, and patient satisfaction
Study	Original, comparative studies, either prospective or RCTs (retrospective studies of prospective data were deemed ineligible)

Other etiologies, such as trauma or revision surgery, were excluded. Studies were allowed to perform accompanying interventions to the lesser metatarsals, such as Weil osteotomy/DMMO or toe deformity corrections.

### Study selection and data extraction

The resulting datasets of each database were exported to EndnoteTM (Vs. 20.1; Fa. Clarivate) and duplicates were removed according to their standard algorithm. The final dataset was imported into CovidenceTM (Melbourne, Australia), in which the complete study selection process was conducted.

Two intendent reviewers (SE, SFB) conducted the whole study selection and data extraction process. In case of disagreement at the stage of title/abstract screen, the studies were moved to full-text screen. Disagreement at the stage of full text-search was resolved by discussion with a third reviewer (HP).

Table 2 shows the data extracted from all qualified primary studies which were recorded on separate data extraction sheets. For the radiologic and clinical data, these were assessed for each time point presented. Radiographic measurements must have been conducted on weightbearing radiographs.

**Table 2:** Data extraction

General information	Location and type of osteotomy, grouping (i.a.), any additional surgical procedures (such as Akin, etc.), postoperative treatment protocol, number of patients and procedures, age, BMI, gender distribution, mean follow-up time
Radiological data	Any established radiological method to examine hallux valgus, i.e. IMA, HVA, DMAA, etc.
Clinical data	Any assessed PROM
Complications	Total amount of complications, complication rate (i.a. classification per minor / major) and complications necessitating further surgical intervention

*IMA* intermetatarsal angle, *HVA* hallux valgus angle, *DMAA* distal metatarsal articular angle, *PROM* patient reported outcome measurement, *i.a.* if applicable

### Risk of bias assessment

The risk of bias assessment was conducted by two independent reviewers (FTS, SFB). For original studies, the level of evidence was rated per the recommendations of Wright et al. [17]. RCTs were assessed per the The Risk of Bias 2 (RoB 2) tool [18]. In case of a non-randomized prospective study, the Newcastle–Ottawa scale was used to assess risk of bias [19].

### Study analysis Jan 1st 2012–Jan 31st 2023

The aim was to perform a meta-analysis to compare the efficacy of different treatment strategies for hallux valgus surgery. Efficacy was defined as the osseous correction and the patient rated outcome per the individual surgical procedures. A multi-step study selection process was performed to identify those studies eligible for a meta-analysis.

First, all studies were grouped per their primary comparator, i.e. comparison of two different surgical procedures or different severities. Then all studies were pooled per their surgical procedures. These were categorized per the anatomical location into open distal, shaft, proximal, or arthrodesis and MIS procedures. The individual studies within each category were then assessed for sufficient comparability. Studies were considered sufficiently comparable if the surgical procedure followed similar biomechanical principles. The studies were transferred into a single data extraction Excel-sheet (Vs. 16.73, Microsoft, Redmond, Washington, USA), listing all radiographic and patient rated outcome measures assessed per the individual studies. For the radiological as well as the patient rated outcome parameters, the time of evaluation was noted.

Only studies comparing different surgical procedures were eligible for a further meta-analysis. Studies comparing different degrees of severity were excluded as the principal grouping resembles a selection bias for the further meta-analysis. The principle meta-analysis was done for all studies per the different surgical techniques. In case of follow-up studies, the initial study was included in the meta-analysis. For the osseous correction**,** no baseline evaluation was performed as only RCTs and prospective studies were included, which most often defined the degree of deformity as an inclusion criterion. This selection bias limits the significance of the degree of preoperative deformity per the different surgical procedures. Therefore, the osseous correction was defined as the difference between the initial and follow-up values. This was done for all radiographic parameters assessed and separated for the type of surgical procedure and follow-up period. Furthermore, any patient rated outcome measure of any other objective outcome parameter was assessed, if presented in at least three studies.

Finally, a meta-regression was performed to investigate the influence of the follow-up duration and initial deformity (IMA, HVA) on the primary outcome parameters, i.e. improvement of the IMA, HVA, and AOFAS score.

### Data synthesis and statistics

Statistical analyses were performed using R 4.05 and the package “meta” version 5.6 to estimate the pooled pre-post differences for IMA, HVA, DMAA, and AOFAS. Heterogeneity among studies was assessed using *I*^2^ test. Effect sizes were compared by osteotomy applying the random effects model. Individual and pooled effect sizes with associated 95% CI were displayed in Forest plots. Studies without SD were excluded from pooled calculations, but were included in forest plots. Confidence intervals for individual studies are based on t-distributions. Missing baseline and final mean and SD data were calculated by approximation according to Luo et al. and Shi et al., respectively, as recommended by Cochrane [20, 21]. To minimize bias, change from baseline SD were imputed assuming a rather conservative correlation between baseline and final values of *r* = 0.4, however, results were confirmed in a sensitivity analysis with *r* = 0.75. Subgroup analysis and meta-regression analyses were conducted to investigate the source of variability between studies. This included follow-up time and baseline values. The presence of publication bias was visually assessed by Funnel plots to measure the asymmetry quantitatively with Egger’s test assuming *p* values less than 0.05 as significant publication bias. Trim-fill analysis was applied to adjust for potential publication bias.

## Results

### Search

The study selection process is outlined in Fig. 1. After removal of duplicates, a total of 3022 studies were screened for title and abstract and 378 for full-text. 46 primary studies [22–67] met the herein defined eligibility criteria. 40 studies [22, 24–35, 37–41, 43, 45–48, 50–57, 59–67] compared different surgical procedures and six studies [23, 36, 42, 44, 49, 58] the same surgical intervention for different severities of hallux valgus deformity.

**Fig. 1:**
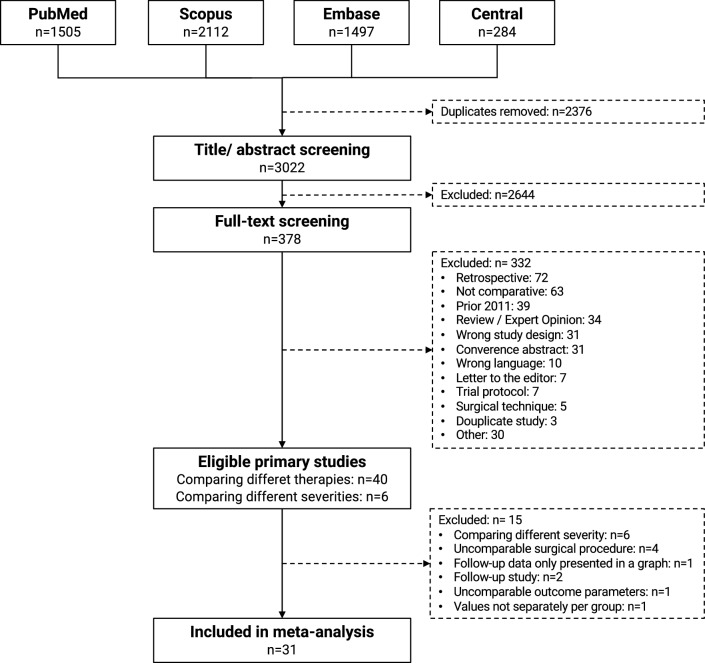
PRISMA flow chart

### Studies overview

Theses 46 studies (100 arms) [22–67] were assessed for their suitability to be included in the meta-analysis (Fig. 2). The cohort consisted of 40 studies comparing different surgical therapies [22, 24–35, 37–41, 43, 45–48, 50–57, 59–67], 30 of which were RCTs [22, 24, 25, 27–31, 33, 34, 37, 39, 40, 46–48, 50–56, 59, 61, 62, 64–67]. Ten studies were prospective comparative cohort or matched group analyses [26, 32, 35, 38, 41, 43, 45, 57, 60, 63], and six studies compared the same surgical procedure at varying degrees of hallux valgus severity [23, 36, 42, 44, 49, 58], all of which were prospective studies. The risk of bias assessment, separate for RCTs (ROB2) and non-randomized prospective trials (Newcastle-Ottawa-Scale), is presented in Supplement 2. Overall, no RCT had a low, 28 RCTs a moderate [22, 24, 25, 28–31, 33, 34, 37, 39, 40, 46, 47, 50–56, 59, 61, 62, 64–67], and two studies a high risk of bias [27, 48]. The Newcastle-Ottawa-Scale per the 16 prospective non-randomized studies resulted in a mean total quality score 6 ± 1 equaling moderate risk of bias [23, 26, 32, 35, 36, 38, 41–45, 49, 57, 58, 60, 63]. Overall, 23 studies (33 arms) included at least one arm with an open distal Chevron osteotomy [25, 28, 29, 36, 37, 39, 40, 42, 43, 45, 46, 50, 51, 53–56, 58–61, 65, 67], 16 studies (23 arms) an open Scarf osteotomy [22, 24, 29, 32, 33, 37, 44, 47, 49–52, 57, 60, 62, 64], three studies (3 arms) an open proximal Chevron osteotomy [34, 46, 55], four studies (5 arms) an open TMT I fusion [30, 31, 35, 43]. Six studies (6 arms) included a 2nd generation MIS [33, 39, 40, 53, 54, 61], and seven studies (9 arms) a 3rd generation MIS procedure [26, 28, 32, 38, 41, 47, 64], and 11 studies (16 arms) other, incomparable procedures for hallux valgus [22, 23, 25, 27, 30, 31, 34, 62, 63, 65, 66]. One study group [32] was contacted throughout the review process, as they did not report on the actual follow-up period. The authors stated to have a mean follow-up of two years, however they could not provide the mean ± SD. Therefore, the follow-up was defined as 2 years.

**Fig. 2:**
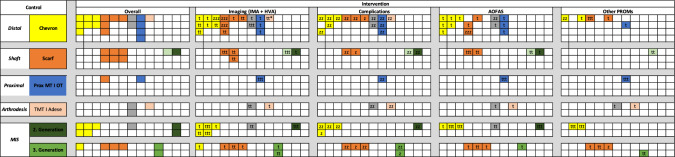
Overview of the eligible studies. Yellow: Open Chevron osteotomy; Grey: Other open distal osteotomies; Orange: Open Scarf osteotomy; Red: Other open shaft osteotomies; Blue: Proximal first metatarsal osteotomies; Salmon: Open first tarso-metatatarsal arthrodesis; Dark green: Second generation MIS; Light green: Third generation MIS (MICA, PECA, MIS Scarf); t: Preoperative/Final follow-up; tt: Pre/Postoperative; ttt: preoperativ/postoperative/Final follow-up; zzz: Preoperative; z: Postoperative; zz: Final follow-up; *: incomplete

### Meta-analysis

Per the above outlined criteria, 31 studies, comprising of 53 study arms were included in the final meta-analysis [22, 24–26, 28–30, 32–35, 37–39, 41, 43, 45–47, 50, 52–56, 60–62, 64, 65, 67]. Excluded were all six studies comparing different severity [23, 36, 42, 44, 49, 58], four studies on surgical procedures incomparable to the remaining procedures [27, 48, 63, 66], two follow-up studies [31, 40], and one study each that presented the outcome data only in a graph [51], assessed incomparable outcome parameters [57], or did not report outcomes separately for the groups [59]. Of the resulting 31 studies, one study arm had to be excluded in eight studies because of incomparable surgical procedures [22, 25, 29, 30, 34, 50, 62, 65].

The IMA, HVA, and the AOFAS were the only outcome parameters assessed by at least three studies. The IMA/HVA/AOFAS were assessed preoperative and at some point postoperatively by 29 studies [22, 24–26, 28–30, 32–35, 37–39, 41, 45–47, 50, 52–56, 61, 62, 64, 65, 67] (49 arms)/30 studies (51 arms) [22, 24–26, 28–30, 32–35, 37–39, 41, 43, 45–47, 50, 52–56, 61, 62, 64, 65, 67]/ and 23 studies (41 arms) [22, 30, 32–35, 37–39, 46, 47, 53, 55, 56, 60–62, 64, 65]. These studies were eligible for a further meta-analysis. The authors have also tried to conduct a summative analysis of the complication rates. Due to a high heterogeneity in the definition of minor and major complications, no meaningful analysis could be conducted.

Among the 31 studies included, 23 studies (38 arms) were RCTs [22, 24, 25, 28–30, 33, 34, 37, 39, 46, 47, 50, 52–56, 61, 62, 64, 65, 67], all with moderate risk of bias per the ROB2 tool and eight studies (15 arms) [26, 32, 35, 38, 41, 43, 45, 60] were non-randomized prospective trials with a mean Newcastle-Ottawa-Scale of 6 ± 2 resulting in moderate risk of bias. The patients mean age was 50 ± 9 years, 88 ± 19% were female and the mean follow-up was 34 ± 37 months. The results of the meta-analysis for the IMA (Fig. 3A), HVA (Fig. 3B), and AOFAS (Fig. 3C) are summarized in Fig. 3. The individual forest-plots are presented in Supplement 3. Based on 1933 procedures, the IMA values improved on average by 7.3 (CI 95%: 6.7°; 7.9°), without significant differences between the different osteotomy groups. Based on 1883 procedures, the HVA improved on average by 18.9° (CI 95%: 17.3°; 20.4°). Third MIS generation procedures resulted in a significantly better HVA correction (21.2° (CI 95%: 19.2°; 23.2°) compared to all other procedures. Based on 1338 procedures, the AOFAS score improved on average by 33.8 points (CI 95%: 30.5; 37.0). No significant differences were observed between the different procedures.

**Fig. 3:**
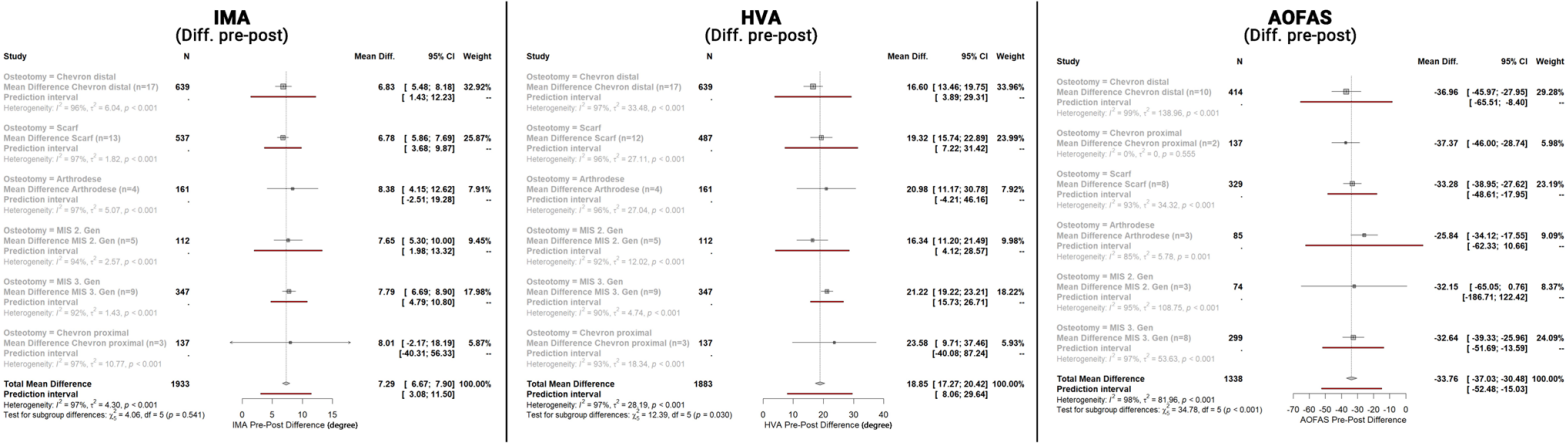
Abbreviated summary of the meta-analysis for the IMA, HVA, and AOFAS. *IMA* intermetatarsal angle, *HVA* hallux valgus angle, *AOFAS* American Orthopaedic Foot and Ankle Society Score

Overall, the meta-analysis revealed substantial heterogeneity between studies and subgroups (> 90%, *p* < 0.001) except for the AOFAS Chevron proximal (*I*^2^: 0%, *p* = 0.555) which included only two studies (see Forest Plots). Funnel plots were symmetric for DMAA (Egger’s test *p* = 0.79) and AOFAS (*p* = 0.065), but were asymmetric for IMA (*p* < 0.0001) and HVA (*p* = 0.03). When the Trim-fill method was applied, the adjusted IMA Pre-Post difference was 5.90° [95% CI 5.16°; 6.63°] (18 additional studies), and the HVA Pre-Post difference was 20.21° [95% CI 18.98°; 22.63°] (9 additional studies). Within the sensitivity analyses, we were also able to confirm the results with higher correlation values between the pre and post values.

### Meta-regression

Finally, the authors tried to assess the influence of the follow-up duration and initial deformity (IMA, HVA) on the primary outcome parameters, i.e. improvement of the IMA, HVA, and AOFAS score.

Firstly, the studies included were analyzed to see if the follow-up duration has an influence on the correction potential (difference pre- and post-operative values of IMA and HVA). Overall, there appears to be only a limited causal relationship, which was predominantly triggered by six studies. The AOFAS score showed no considerable change over time.

Secondly, a possible influence of the preoperative values (IMA, HVA, AOFAS) on the correction potential (difference pre- and post-operative values of IMA and HVA) was assessed. 69%/39% of the correction potential for both, IMA and HVA, could be explained by the respective preoperative values. For the improvement of the AOFAS, 82% could be explained by the preoperative AOFAS score (Fig. 4).

**Fig. 4:**
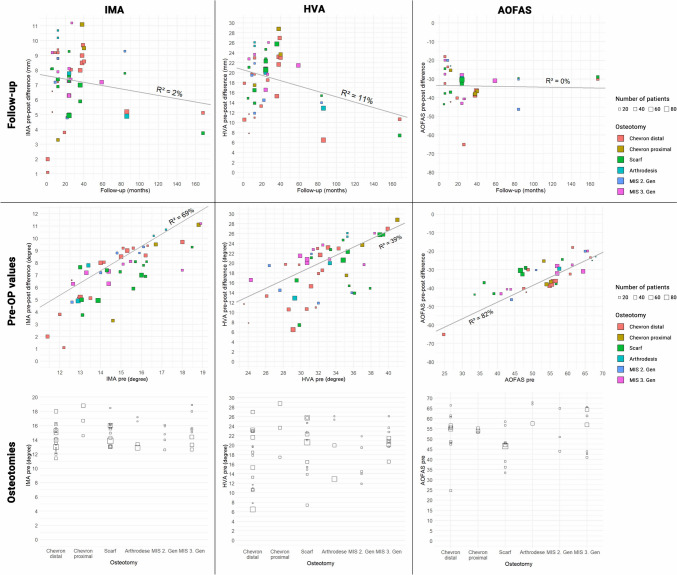
Meta-regression plotted and individual per studies. *IMA* intermetatarsal angle, *HVA* hallux valgus angle, *AOFAS* American Orthopaedic Foot and Ankle Society Score, *pre-OP* preoperative, *MIS* minimal invasive surgery

## Discussion

This systematic review and meta-analysis aimed to develop a “living systematic review” for the outcome of surgically treated hallux valgus, focusing on the osseous correction and the patient rated outcome, separately for the surgical procedure used. The results of this meta-analysis showed that the osseous correction, measured by IMA and HVA, and postoperative clinical outcome, based on the AOFAS score, did not differ significantly for the included surgical procedures, except for significantly higher correction of HVA in 3rd MIS generation procedures. All surgical procedures showed a significant postoperative improvement of the AOFAS score by a mean of 33.8 points, which was well above the MCID (AOFAS 7.9) [68]. The improvement was best explained by the low pre-operative AOFAS scores in patients undergoing surgery for symptomatic hallux valgus.

The severity of the hallux valgus deformity may be classified into ‘mild’, ‘moderate’ and ‘severe’ [1]. Interestingly, the traditional classification proposed by Mann and Coughlin, has not been applied uniformly throughout the studies included herein. The upper limits of the IMA for mild/moderate/severe ranged between 9° [54]/12° [47]/17° [55, 56] and 15° [45]/20° [27, 29, 50, 65]/20° [29, 50, 54] degrees. This limits the comparability between studies. Journals and authors should agree on a uniform definition for hallux valgus deformities. With novel imaging modalities emerging, a uniform definition should also include the distal metatarsal articular angle and the rotation of the first metatarsal, to more clearly define the pathology.

The preoperative IMA/HVA differed widely between each procedure. Contrary to expectations, a correlation between the preoperative IMA/HVA and the surgical procedure, i.e. more proximal techniques for more severe deformities, could not be shown. Since no differences in the osseous correction potential per the different surgical procedures could be detected, the question arises whether the correction potential of the presumably more powerful, proximal techniques was underestimated. One reason could be the inclusion of less severe deformities into this group as evidenced by missing correlation between preoperative angles and surgical procedure. Secondly, a source of bias could be the number of studies included. A substantially lower number of studies reported on TMT 1 arthrodesis (*n* = 3; arms: *n* = 4; patients: *n* = 161) patients), compared to distal chevron- (*n* = 14; arms: *n* = 17; patients: *n* = 639), proximal chevron (*n* = 3; arms: *n* = 3; patients: *n* = 137), scarf-osteotomy (*n* = 11; arms: *n* = 13; patients: *n* = 537), MIS^2nd^ generation (*n* = 5; arms: *n* = 5; patients: *n* = 161) and MIS 3rd generation (*n* = 7; arms: *n* = 9; patients: *n* = 347). Thirdly, due to the small number of studies included in the arthrodesis group, a single study reporting inferior outcomes has a pronounced effect on the final result, which was the case for the study of Klemola et al. [43]. Finally, the choice of the surgical procedure cannot be solely based on the preoperative IMA and HVA. Factors such as instability of the medial column, severe rotational malalignment of the MT I and TMT I arthritis represent main indications for a TMT 1 arthrodesis [69–71].

Over the last decade there is a trend towards minimally invasive hallux valgus surgery [10, 72, 73]. In our meta-analysis, 3rd MIS generation showed significantly more correction of HVA compared to the other techniques, whereas there were no differences in the correction of IMA and improvement of AOFAS scores. The 2nd MIS generation did not show improved clinical or radiological outcomes compared with the open techniques. This may be due to the fact, that the 3rd MIS generation always includes an akin osteotomy, which has a profound influence on the HVA. An akin procedure was not regularly performed in the included studies with open procedures. A subgroup analysis solely including open procedures with an akin osteotomy was not possible due to missing data. This could bias the results of better HVA correction compared to open procedures. One current meta-analysis compared open versus minimally invasive hallux valgus surgery, including 22 studies, of which eight were RCTs [74]. IMA, HVA, DMAA and the AOFAS score were assessed. Including all 22 studies, there was no significant difference in clinical and radiological outcomes between open and MIS hallux valgus surgery. However, in the subgroup analysis the 2nd MIS generation showed significantly lower postoperative IMA, the 3rd MIS generation significantly lower postoperative HVA compared to open techniques. The AOFAS score was significantly higher in the MIS group, when only RCTs were included. The authors concluded that MIS was more effective than open surgery in the treatment of hallux valgus. When looking at the data presented here, the mid- and long-term results of further RCTs must be awaited before suggesting any superiority of minimally invasive hallux valgus surgery.

The performed meta-regression analysis revealed a tendency towards loss of correction for the IMA and HVA with increasing follow up period, independent of the surgical procedure. However, the AOFAS score was not affected by the follow up duration. The average degree of IMA and HVA correction resulted in a significant improvement of the AOFAS, well above the MCID. Overall, the number of studies with longer follow up was significantly lower, so that only a certain trend could be observed. Similar results were presented in a meta-analysis by Kaufmann et al. comparing MIS distal chevron and MIS reverdin isham osteotomies [41]. In this study, a longer follow up period resulted in a radiological loss of correction (IMA;HVA), which did not influence the AOFAS score. Hence, a certain loss of correction over time might not have a significant impact on the clinical outcome in terms of the AOFAS score.

Overall, each meta-analysis revealed a significant heterogeneity for each parameter assessed. There appears to be a rather broad correction range for each osteotomy individually. This could be due to the initial deformity or the individual surgeons’ skills.

From the visual analysis of the Funnel plots, it appears that the pooled IMA correction potential presented here may have been overestimated because a substantial number of studies with lower pre-post difference for symmetry are missing. Conversely, the pooled HVA correction potential may have been underestimated because studies with higher HVA correction values are missing.

Only comparative studies published after 2012 were included. The number of studies dealing with hallux valgus correction, i.e. single arm studies, is immense and was not included into the current systematic review. Due to a low methodological quality, many studies were excluded for final analysis. As a result, the number of included studies per surgical procedure varies considerably.

In the meantime, indication for surgery is not solely based on traditional radiographic parameters such as IMA and HVA. Simultaneous derotation of the first ray is recognized as one of the key elements in treating hallux valgus to reduce the risk of recurrence [73, 75–78]. Many studies continue to assess only IMA and HVA and do not include metatarsal rotation. Additionally, the DMAA should be measured to detect a pathological joint line, as this significantly influences the recurrence rate [79]. In this meta-analysis, evaluation of the DMAA per surgical procedure was not possible due to the small number of studies including DMAA measurements. The bony morphology must also be considered, since the width of the first metatarsal affects the possibility of maximum translation. Further studies should include these parameters, in order to enable a more differentiated analysis.

Another limitation is the fact that the surgical techniques may be substantially modified by the individual surgeon based on personal experience and preference (e.g. orientation of osteotomy, extent of lateral soft tissue release). This makes comparison of the same technique between different studies difficult.

Another concern is a missing standardized categorization of postoperative complications into minor and major ones. The data of complications in the included studies differ widely—in some studies recurrence was specified as a complication, in others it was not. Therefore, an evaluation of complications depending on the surgical procedure could not be assessed. Recently, it has been tried to establish a modified Clavien-Dindo classification, which does allow for a considerably more detailed assessment of complications than the traditional grouping into minor and major complications [80]. Such a classification should be defined and regularly applied to enable comparability between surgical procedures in the future.

## Conclusion

According to the current evidence, both open and minimally invasive techniques are powerful tools to correct hallux valgus deformity with significantly improved radiological and clinical outcome parameters. Third generation MIS procedures revealed a possible superiority for the correction of the HVA without measurable impact of outcome. Independent of the surgical technique applied, there is a considerable improvement of the AOFAS score which appears to be constant over time, while there is a tendency for loss of correction of the radiologic parameters. We as a community must define uniform reporting strategies for diagnosis, classification, and outcome to increase the comparability between studies.

## Supplementary Information

Below is the link to the electronic supplementary material.

Supplementary file1 (DOCX 14 KB)

Supplementary file2 (XLSX 28 KB)

Supplementary file3 (PDF 448 KB)

## Data Availability

Data can be shared if necessary.

## Declarations

### Copyright/license for publication

Granted.
