# Supplementary material for: Correction potential and outcome of various surgical procedures for hallux valgus surgery: a living systematic review and meta-analysis
Source: Arch Orthop Trauma Surg. 2024 Sep 9;144(11):4725–36. doi: 10.1007/s00402-024-05521-0 (PMC11582212; doi:10.1007/s00402-024-05521-0)
Supplement: Supplementary file 1 — Supplementary file1 (DOCX 14 KB) [file 402_2024_5521_MOESM1_ESM.docx]

MEDLINE (PubMed)

("hallux valgus"[MeSH Terms] OR "hallux valgus"[Title/Abstract] OR “Hallux Abductovalgus"[Title/Abstract] OR "hallux-valgus"[Title/Abstract]) AND ("surgery"[MeSH Subheading] OR "surgical"[Title/Abstract] OR "surgery"[Title/Abstract] OR "operative*"[Title/Abstract] OR "operation"[Title/Abstract] OR "repair"[Title/Abstract] OR "repaired"[Title/Abstract] OR "arthrodesis"[MeSH Terms] OR "arthrodesis"[Title/Abstract] OR "arthrodeses" [Title/Abstract] OR "Osteotomy"[Mesh] OR "Osteotomy"[Title/Abstract] OR "Osteotomies"[Title/Abstract]) AND ("2012/01/01"[Date - Entry] : "2021/04/01"[Date - Entry])

Scopus

TITLE-ABS-KEY ("hallux valgus" OR “Hallux Abductovalgus") AND TITLE-ABS-KEY ("surgery" OR "surgical" OR "operative*" OR "operation" OR "repair" OR "repaired" OR "arthrodesis” OR "arthrodeses" OR "Osteotomy" OR "Osteotomy" OR "Osteotomies") AND ORIG-LOAD-DATE AFT "2012/01/01"( ( TITLE-ABS-KEY ( hallux AND valgus ) OR TITLE-ABS-KEY ( hallux AND abductovalgus ) OR TITLE-ABS-KEY ( hallux-valgus ) ) AND ( TITLE-ABS-KEY ( surgery ) OR TITLE-ABS-KEY ( surgical ) OR TITLE-ABS-KEY ( operative* ) OR TITLE-ABS-KEY ( operation ) OR TITLE-ABS-KEY ( repair ) OR TITLE-ABS-KEY ( repaired ) OR TITLE-ABS-KEY ( arthrodesis ) OR TITLE-ABS-KEY ( arthrodeses ) OR TITLE-ABS-KEY ( osteotomy ) OR TITLE-ABS-KEY ( osteotomy ) OR TITLE-ABS-KEY ( osteotomies ) ) ) AND ( LIMIT-TO ( PUBYEAR , 2021 ) OR LIMIT-TO ( PUBYEAR , 2020 ) OR LIMIT-TO ( PUBYEAR , 2019 ) OR LIMIT-TO ( PUBYEAR , 2018 ) OR LIMIT-TO ( PUBYEAR , 2017 ) OR LIMIT-TO ( PUBYEAR , 2016 ) OR LIMIT-TO ( PUBYEAR , 2015 ) OR LIMIT-TO ( PUBYEAR , 2014 ) OR LIMIT-TO ( PUBYEAR , 2013 ) OR LIMIT-TO ( PUBYEAR , 2012 ) )

Central

(MeSH descriptor: [hallux valgus] OR "hallux valgus":ti,ab,kw OR “Hallux Abductovalgus":ti,ab,kw OR "hallux-valgus":ti,ab,kw) AND (MeSH descriptor: ["surgery"] OR "surgical":ti,ab,kw OR "surgery":ti,ab,kw OR "operative*":ti,ab,kw OR "operation":ti,ab,kw OR "repair":ti,ab,kw OR "repaired":ti,ab,kw OR MeSH descriptor: ["arthrodesis"] OR "arthrodesis":ti,ab,kw OR "arthrodeses":ti,ab,kw OR MeSH descriptor: ["Osteotomy"] OR "Osteotomy":ti,ab,kw OR "Osteotomies":ti,ab,kw AND limit #N to yr=2012-2021

EMBASE

(exp hallux valgus OR ‘hallux valgus’:ti,ab OR ’Hallux Abductovalgus’:ti,ab OR ‘hallux-valgus’:ti,ab) AND (exp ‘surgery’ OR ‘surgical’:ti,ab OR ‘surgery’:ti,ab OR ‘operative*’:ti,ab OR ‘operation’:ti,ab OR ‘repair’:ti,ab OR ‘repaired’:ti,ab OR exp ‘arthrodesis’ OR ‘arthrodesis’:ti,ab OR’arthrodeses’:ti,ab OR exp ‘Osteotomy’ OR ’Osteotomy ’:ti,ab OR ‘Osteotomies’:ti,ab) AND [2012-2021]/py
