# Supplementary figures and images for: Correction potential and outcome of various surgical procedures for hallux valgus surgery: a living systematic review and meta-analysis
Source: Arch Orthop Trauma Surg. 2024 Sep 9;144(11):4725–36. doi: 10.1007/s00402-024-05521-0 (PMC11582212; doi:10.1007/s00402-024-05521-0)

IMA  
(Diff. pre-post)

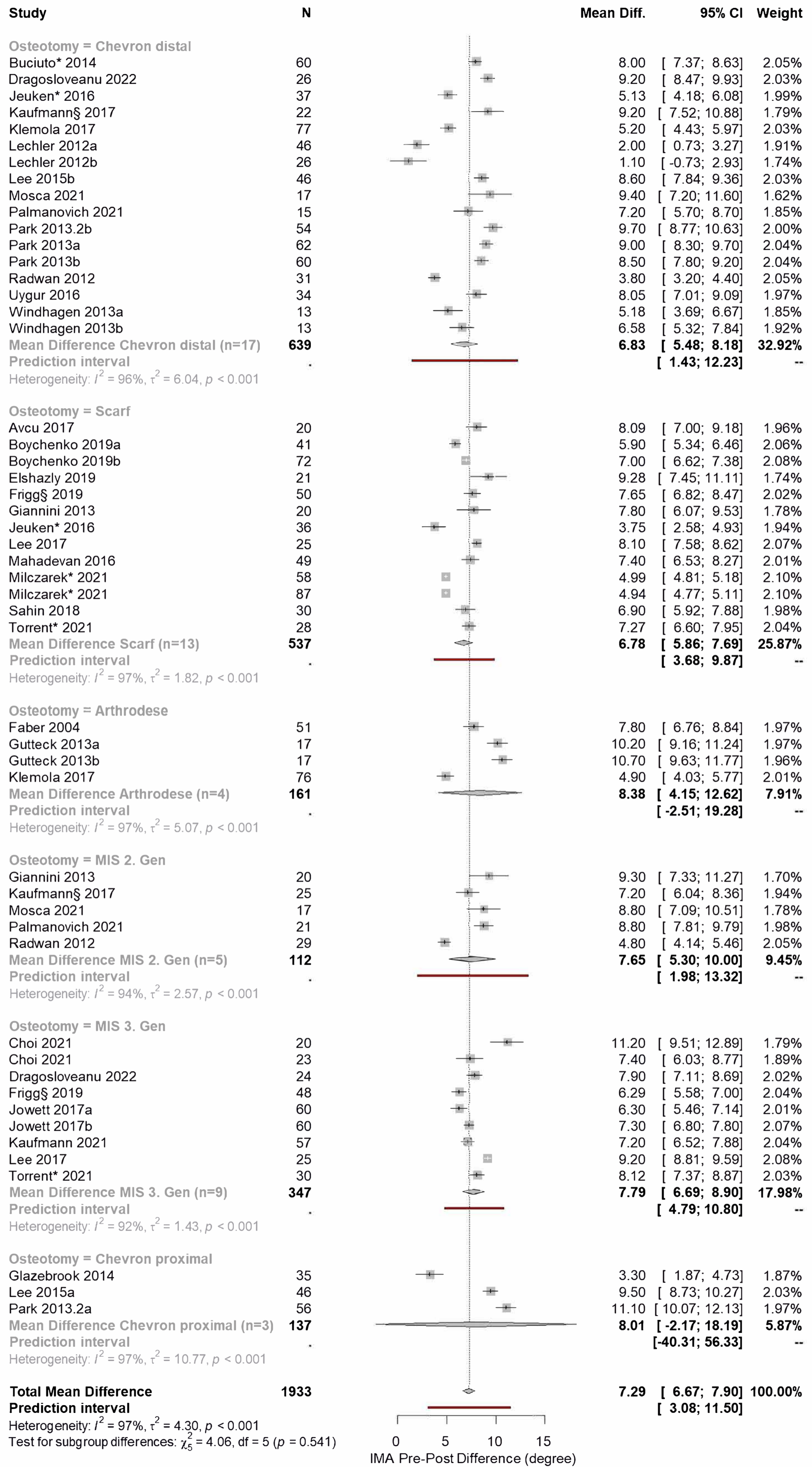

HVA  
(Diff. pre-post)

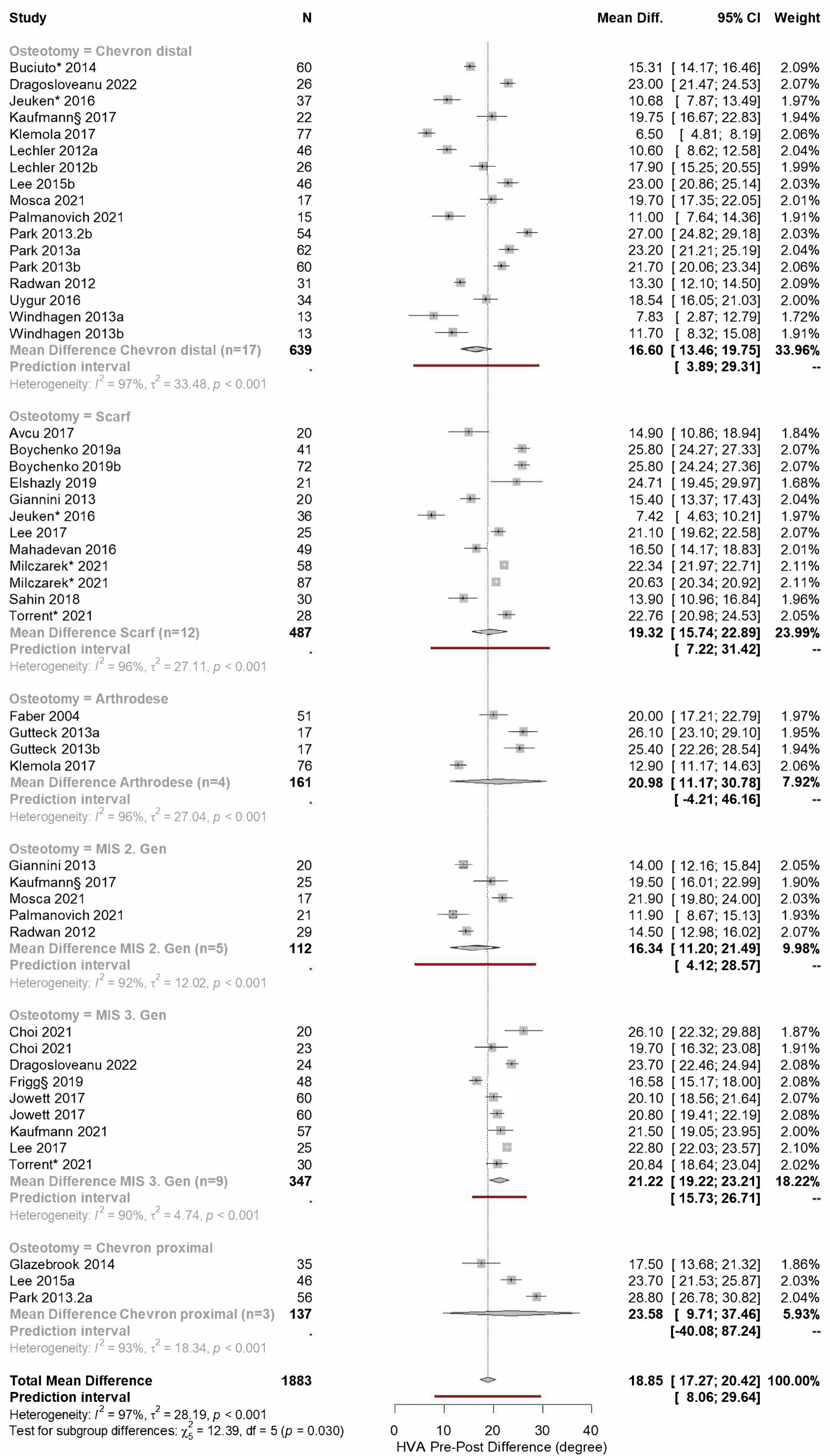

# AOFAS

(Diff. pre-post)

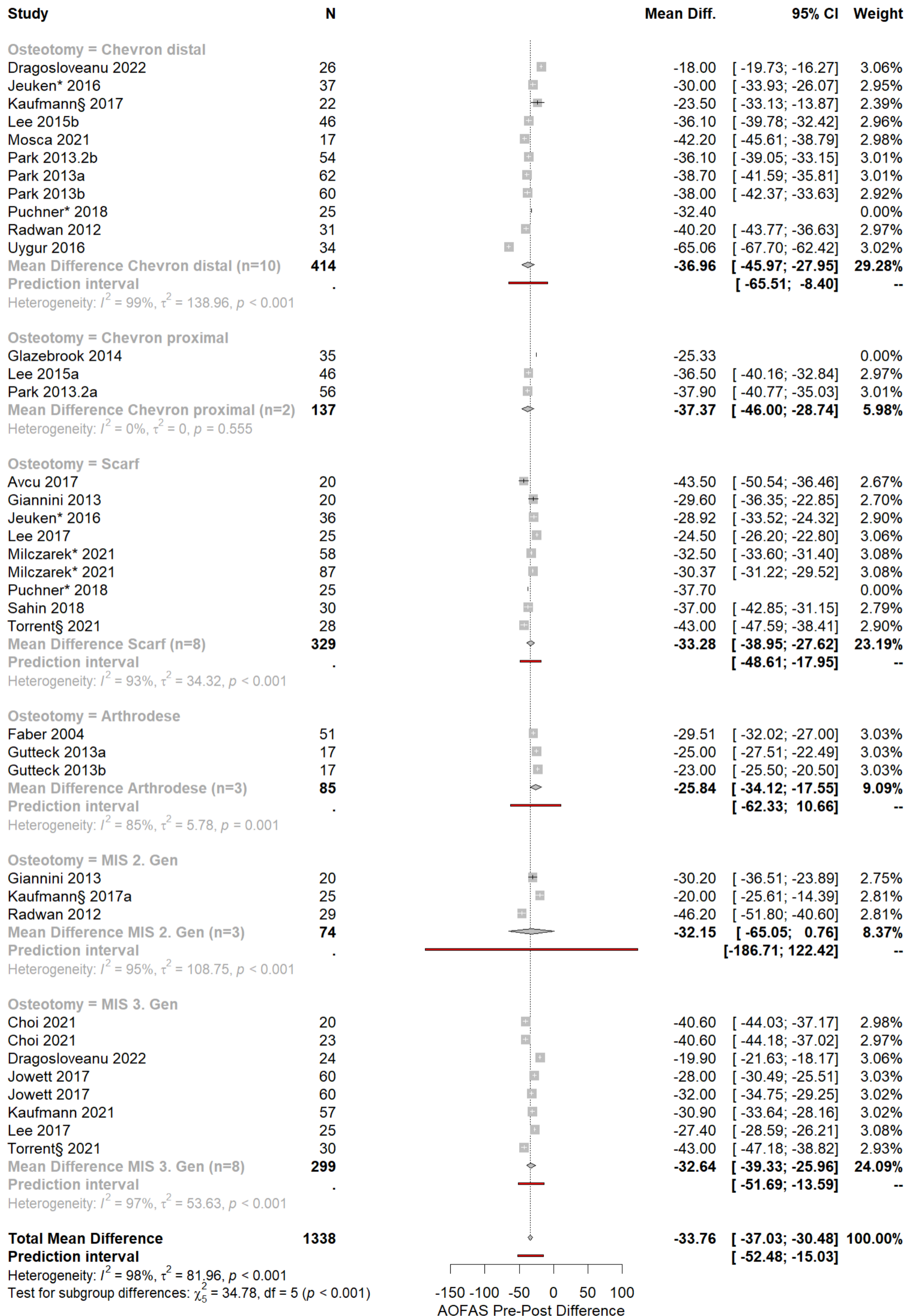

Supplement: Supplementary file 3 — Supplementary file3 (PDF 448 KB) [file 402_2024_5521_MOESM3_ESM.pdf]
